# *In vitro* antibacterial activity of *Piper betel* extract nanoemulsion and cannabidiol formulations against methicillin-resistant *Staphylococcus* isolates from canine pyoderma

**DOI:** 10.14202/vetworld.2025.3017-3028

**Published:** 2025-10-14

**Authors:** Wongsakorn Wongwatcharamongkhon, Pareeya Udomkusonsri, Natthasit Tansakul, Udomlak Sukatta, Suporn Thongyuan, Watsapol Suntoranan, Chantima Pruksakorn

**Affiliations:** 1 Department of Microbiology and Immunology, Faculty of Veterinary Medicine, Kasetsart University, 50 Ngamwongwan Rd., Bangkok 10900, Thailand; 2 Department of Pharmacology, Faculty of Veterinary Medicine, Kasetsart University, 50 Ngamwongwan Rd., Bangkok 10900, Thailand; 3 Kasetsart Agricultural and Agro-Industrial Product Improvement Institute, Kasetsart University, Bangkok 10900, Thailand; 4 Department of Veterinary Public Health, Faculty of Veterinary Medicine, Kasetsart University, Nakhon Pathom, Thailand

**Keywords:** antimicrobial resistance, canine pyoderma, cannabidiol, nanoemulsion, *Piper betel*, *Staphylococcus pseudintermedius*

## Abstract

**Background and Aim:**

Canine pyoderma is a common dermatological condition, often caused by *Staphylococcus pseudintermedius* and related methicillin-resistant strains (MRSP and MRSS). Rising antimicrobial resistance necessitates alternative topical therapies. This study comparatively evaluated the *in vitro* antibacterial activity of *P. betel* leaf extract (both solvent-based and nanoemulsion forms) and cannabidiol (CBD) formulations against canine *Staphylococcus* isolates.

**Materials and Methods:**

Antibacterial activity was determined by broth microdilution to establish minimum inhibitory concentration (MIC) and minimum bactericidal concentration (MBC). Five formulations were tested: Ethanolic betel leaf extract in dimethyl sulfoxide betel leaf (BL), BL extract nanoemulsion (BLN), CBD in ethanol, water-soluble CBD, and CBD nanoemulsion. Test organisms included 15 *Staphylococcus* isolates (five MRSP, five methicillin-susceptible *S. pseudintermedius*, and five MRSS) and five *Pseudomonas aeruginosa*. Gas chromatography-mass spectrometry (GC-MS) was used to characterize phytochemical constituents.

**Results:**

GC-MS revealed eugenol (40.86%) and hydroxychavicol (26.44%) as predominant antibacterial compounds. BL and BLN demonstrated potent anti-staphylococcal activity, with median MICs of 0.16 g/L and 0.31 g/L, respectively. BL exhibited significantly lower MIC and MBC values than BLN (p = 0.008). Among CBD formulations, ethanol-dissolved and water-soluble CBD displayed the strongest activity (median MICs 0.003 g/L and 0.004 g/L), while CBD nanoemulsion was markedly less effective (median MIC 7.50 g/L). BLN also exhibited antibacterial activity against *P. aeruginosa* (median MIC 0.62 g/L), comparable to BL.

**Conclusion:**

The novel BLN and soluble CBD formulations demonstrated significant *in vitro* antibacterial activity against methicillin-resistant *Staphylococcus* isolates from canine pyoderma. These results highlight their potential as topical antiseptic alternatives to chlorhexidine. Further *in vivo* studies are required to assess safety, efficacy, and formulation optimization. A combined betel-CBD nanoemulsion represents a promising direction for developing novel veterinary dermatological therapies.

## INTRODUCTION

Pyoderma is a common infectious skin disease in dogs that produce distressing symptoms such as itching and pain [1]. Epidemiological estimates indicate that it affects 1.25%–2.67% of the general canine population and up to 11% of dogs presenting with dermatological conditions [2–5]. The disorder is typically bacterial and secondary in nature, often triggered by predisposing factors including allergies, hypothyroidism, or fungal infections [1]. *Staphylococcus pseudintermedius* is the predominant bacterial species isolated, accounting for up to 92% of cases, and occurs in both methicillin-resistant *S. pseudintermedius* methicillin-resistant (MRSP) and methicillin-susceptible (MSSP) forms [6]. Other pathogens, such as *Staphylococcus schleiferi* and certain Gram-negative bacteria, are less frequently involved [1, 7, 8].

The growing threat of antimicrobial resistance (AMR) poses a significant challenge under the One Health framework, which links human, animal, and environmental health [9]. Multidrug-resistant strains, particularly MRSP and methicillin-resistant *Staphylococcus schleiferi* (MRSS) subsp. *coagulans*, have emerged as major concerns in veterinary medicine, severely limiting therapeutic options [10, 11]. Prevalence study by Beck *et al*. [8] reports MRSP in 40.5% of canine pyoderma cases and MRSS in 2.9%. The increasing resistance of *S. pseudintermedius* is alarming, and some methicillin-resistant strains also pose zoonotic risks [12]. These findings highlight the urgent need for new antiseptic and disinfection strategies aligned with the One Health concept [1, 6, 13, 14].

Topical antibacterial therapy remains a cornerstone in the management of canine pyoderma. It may be applied as monotherapy for localized infections or combined with systemic antimicrobials in more severe cases [15]. Long-term topical therapy is also recommended to prevent relapse and slow the development of AMR [15, 16]. Conventional antiseptics include chlorhexidine, benzoyl peroxide, ethyl lactate, and acetic acid [16]. Chlorhexidine is widely used due to its well-documented *in vivo* antibacterial efficacy [17]; however, its prolonged or excessive application can induce side effects such as pruritus and erythema [18]. Moreover, reports of reduced susceptibility to chlorhexidine reinforce the demand for novel antimicrobial agents, especially those derived from natural sources [17].

Medicinal plants are increasingly recognized as promising alternatives for infection control in veterinary medicine [19]. Their diverse phytochemical compositions provide broad-spectrum antimicrobial activity, and they are generally considered to have a lower potential for inducing resistance [19, 20].

*Piper betel* L. (locally known as “Phlu” in Thailand) exemplifies such potential. Conventionally used across India and Southeast Asia, it is rich in flavonoids and phenols with established antimicrobial properties [21, 22]. Previous research by Phensri *et al*. [23] demonstrated that betel leaf extract exhibits potent *in vitro* antibacterial activity against canine staphylococcal isolates, including methicillin-resistant strains. Notably, its minimum inhibitory concentration (MIC) was significantly lower than that of benzoyl peroxide, suggesting strong potential for development as a topical treatment for canine pyoderma.

Cannabis (*Cannabis sativa* L.) derivatives, particularly cannabidiol (CBD), are also attracting growing interest in veterinary therapeutics [24]. Among the more than 120 phytocannabinoids identified, CBD is preferred for medical applications due to its lack of psychoactive effects, unlike tetrahydrocannabinol [25]. CBD has demonstrated antibacterial efficacy against methicillin-resistant *Staphylococcus aureus* [26] and antibacterial as well as anti-biofilm activity against *Streptococcus mutans* [27]. However, its effectiveness against clinically relevant canine isolates such as MRSP and MRSS remains underexplored, warranting further investigation.

Despite their potential, approximately 90% of natural products face limitations, including poor water solubility, restricted bioavailability, and low stability, which hinder their clinical efficacy [28]. For instance, both betel leaf extract and CBD suffer from limited solubility, reducing their therapeutic potential unless optimized with advanced delivery systems [29, 30]. Furthermore, penetration of natural compounds through the stratum corneum remains a barrier in *in vivo* applications [31, 32]. Nanoemulsions, with their submicron droplet size and high colloidal stability, represent a promising strategy to overcome these limitations. They enhance solubility, improve dermal absorption, and increase antimicrobial potency by facilitating better delivery of lipid-soluble compounds [33, 34]. For example, nanoemulsions of bay leaf extract and clove essential oil demonstrated superior antibacterial activity against *S. aureus* and *Escherichia coli* compared with unformulated extracts [35, 36]. These findings underscore the value of nanoemulsions in enhancing the therapeutic potential of natural products.

Taken together, the shortcomings of current topical therapies and the limitations of natural agents underscore a critical research gap. Although the antibacterial potential of *Piper betel* and CBD has been individually documented, no natural formulation has yet proven clinically effective against MRSP or MRSS in veterinary medicine. Challenges such as achieving effective therapeutic concentrations without toxicity and the lack of standardized, controlled veterinary trials demonstrating clinical efficacy and safety continue to impede progress [37].

Despite the growing recognition of AMR in veterinary dermatology, particularly in canine pyoderma caused by MRSP and MRSS subsp. *coagulans*, treatment options remain limited. Conventional topical antiseptics such as chlorhexidine, benzoyl peroxide, and acetic acid are increasingly associated with adverse effects, reduced susceptibility, and patient compliance issues. Medicinal plants, such as *P. betel* and bioactive compounds such as CBD have demonstrated promising antibacterial properties *in vitr*o, yet their clinical translation has been hindered by poor solubility, low bioavailability, and insufficient dermal penetration. Although nanoemulsion-based delivery systems have been shown to enhance antimicrobial activity of natural compounds, no previous study has systematically compared betel leaf extract nanoemulsion with different CBD formulations against clinically relevant canine staphylococcal isolates. Moreover, their combined potential as natural, topical alternatives to synthetic antiseptics remains unexplored. This lack of comparative evaluation and formulation optimization constitutes a critical knowledge gap in the search for safe, effective, and sustainable topical therapies for canine pyoderma.

The present study aimed to evaluate and compare th*e in vitro* antibacterial activity of betel leaf extract (in solvent-based and nanoemulsion forms) and three CBD formulations (ethanol-dissolved CBD, water-soluble CBD, and CBD nanoemulsion) against clinical isolates of MRSP, MSSP, and MRSS from dogs with pyoderma. In addition, the antibacterial potential of betel leaf extract nanoemulsion was tested against *Pseudomonas aeruginosa*, an important Gram-negative pathogen commonly associated with canine skin infections. By determining MIC and minimum bactericidal concentration (MBC) values, this study sought to identify the most effective formulations and establish a scientific foundation for developing plant-based antiseptic alternatives. Ultimately, the goal was to provide evidence for the potential application of *P. betel* and CBD nanoformulations as novel, natural topical agents that may reduce reliance on conventional antiseptics and support antimicrobial stewardship in veterinary medicine.

## MATERIALS AND METHODS

### Ethical approval

This *in vitro* study utilized clinical bacterial isolates obtained from a strain collection. The isolates were collected as part of routine diagnostic procedures and standard veterinary care. No additional interventions were performed on animals specifically for this research; therefore, no specific ethical approval was required.

### Study period and location

The study was conducted between April 2023 and January 2025 at the Department of Microbiology and Immunology, Faculty of Veterinary Medicine, Kasetsart University, Bangkok, Thailand.

### Bacterial strains and culture conditions

For quality control, two reference strains of *Staphylococcus aureus* (American Type Culture Collection [ATCC] 6538, Department of Medical Sciences Thailand [DMST] 8013; ATCC 29213, DMST 4745) were used, obtained from the Department of Medical Sciences, Nonthaburi, Thailand. Clinical isolates from canine pyoderma were retrieved from the culture collection of the Department of Microbiology and Immunology, Kasetsart University. These included:

- 5 MRSP,
- 5 MSSP,
- 5 MRSS subsp. *coagulans*, and
- 5 *Pseudomonas aeruginosa*.

The *Staphylococcus* strains had been previously characterized as methicillin-resistant or methicillin-susceptible [23]. *P. aeruginosa* was confirmed using Matrix-Assisted Laser Desorption Ionization-Time-of-Flight Mass Spectrometry (MALDI-TOF-MS) (Vitek MS, bioMérieux SA, Marcy-l’Étoile, France).

All isolates were stored in Luria-Bertani broth with 20% glycerol (MilliporeSigma, Burlington, MA, USA) at −80°C. Before testing, strains were thawed, subcultured on tryptic soy agar (Becton Dickinson, Franklin Lakes, NJ, USA), and incubated at 37°C for 18–24 h to ensure active growth.

### Preparation of betel leaf extract

Fresh betel leaves were purchased from Talad Thai Market in Pathum Thani Province, Thailand. Plant identity was authenticated by a botanist, and a voucher specimen (C. Pruksakorn 252PD; family: Piperaceae; species: *P. betel* L.) bearing the registration number BK086364 was formally deposited in the Bangkok Herbarium.

The extract was prepared as a crude ethanolic extract [23, 38]. Leaves were washed, cut into small pieces, dried in a tray dryer at 45°C for 24 h, and ground into powder. One hundred grams of dried powder was extracted with 95% ethanol (Merck KGaA, Darmstadt, Germany) 3 times at room temperature (25°C) with 3-day maceration. Filtrates were pooled and concentrated using a rotary evaporator (Buchi Rotavapor R-124, Buchi Labortechnik AG, Flawil, Switzerland) at 40°C, yielding approximately 10% extract.

The percentage yield was calculated as:

Yield (%) = (weight of crude extract/weight of dried leaf) × 100.

### Phytochemical characterization by chromatography-mass spectrometry (GC-MS)

Chemical composition was analyzed using gas GC-MS at the Kasetsart Agricultural and Agro-Industrial Product Improvement Institute. A Shimadzu Nexis GC-2030NX with a DB-5MS capillary column (30 m × 0.32 mm; 0.50 μm coating thickness) and a GC-2030 mass selective detector was used.

Operating conditions: Injector at 280°C, transfer line at 250°C, oven programmed from 80°C (3 min hold) to 250°C at 10°C min^-1^, with a final 4 min hold. Helium served as the carrier gas at 1.49 mL min^-1^. Samples were diluted 2:1000 (v/v) with petroleum ether and injected at 1.0 μL volumes (split ratio 1:20). Mass spectra were acquired in full scan (35–500 m/z) with 70 eV electron impact ionization.

Compounds were identified by comparison with the National Institute of Standards and Technology (NIST) W11N/17M1 library. Relative quantification was performed using percentage peak area.

### Formation and characterization of betel leaf extract nanoemulsion

The base composition of the nanoemulsion is shown in Table 1. To prepare the formulation, 0.5 g of betel extract was dissolved in 5 g of mixed medium-chain fatty acid oils. This mixture was blended with 5 g of mixed surfactants (Tween 80, Span 80, Poloxamer 407 at 2:2:1). Hydrophilic components – 70% sorbitol solution (5 g), propylene glycol (3 g), polyethylene glycol 400 (1 g), paraben concentrate (1 g), and sodium ethylenediaminetetraacetic acid (0.1 g) – were sequentially incorporated to form a pre-emulsion.

**Table 1:** Components of the nanoemulsion base.

Ingredient	Percentage (w/w)
Oil mixture	3–5
Mixed surfactant	3–5
70% sorbitol	3–5
Propylene glycol	1.5–3
Polyethylene glycol 400	1–3
Paraben concentration	0.5–2
Antioxidant	0.05–1
Sodium EDTA	0.05–1

EDTA = Ethylenediaminetetraacetic acid

The pre-emulsion was homogenized at 1,000 rpm for 10 min, followed by high-pressure homogenization (15,000 psi, 5 cycles). Purified water was added to achieve a 100 g final weight. The resulting nanoemulsion was a creamy yellow liquid with a droplet size of 147.43 ± 5.41 nm, polydispersity index of 0.44 ± 0.01, and zeta potential of –1.42 ± 0.16 mV. Centrifugation stability testing confirmed its kinetic stability.

A control extract solution was prepared in 10% dimethyl sulfoxide (DMSO) at 5 g/L, used for comparison in antimicrobial assays.

### Preparation of CBD formulations and nanoemulsions

Three CBD formulations were prepared:

1. CBD in ethanol,
2. Water-soluble CBD formulation, and
3. CBD nanoemulsion.

CBD isolate (99.9% purity, Salus Bioceutical, Thailand) was used for the ethanol and nanoemulsion formulations. A preformulated water-soluble CBD powder (20% CBD with starch excipients) was also obtained. Final concentrations are shown in Table 2.

**Table 2:** Stock concentration of the reagents.

Reagent	Stock concentration of reagents (g/L)
Betel leaf extract in 10% DMSO solution	1.0 and 5.0
Betel leaf extract nanoemulsion	1.0 and 5.0
CBD in absolute ethanol concentration*	1.0
Water-soluble CBD*	20.0
CBD nanoemulsion	30.0

The stock solutions were prepared at a concentration of 2×, except for

* , which was prepared at a concentration of 10×. CBD = Cannabidiol, DMSO = Dimethyl sulfoxide

- Ethanol solution: 10 mg CBD in 10 mL absolute ethanol (1 g/L), stirred at 45°C for 30 min
- Water-soluble CBD: 2 g of preformulated powder dissolved in 20 mL purified water (20 g/L)
- Nanoemulsion: Oil-in-water emulsion prepared at 30 g/L CBD using high-pressure homogenization (15,000 psi, 5 cycles), with droplet sizes of 150–200 nm.

All formulations were visually stable and confirmed for CBD content by high-performance liquid chromatography with diode array detection [39].

### Determination of MIC and MBC

MIC values were determined using the broth microdilution method [23, 40]. Serial dilutions of stock solutions were prepared in cation-adjusted Mueller–Hinton broth and dispensed into 96-well plates.

Bacterial suspensions (10^6^ colony-forming units CFU/mL) were added to wells and incubated at 37°C for 18–24 h. Afterward, 30 μL of 0.01% resazurin was added, and plates were incubated for 2–4 h. MIC was defined as the lowest concentration with no visible growth (blue color). Tests were performed in triplicate with media, solvent, and nanoemulsion blanks as controls.

MBC was determined through spot plating of MIC wells on tryptic soy agar, followed by incubation at 37°C for 18–24 h. The lowest concentration achieving ≥99.9% killing (no colony growth) was considered the MBC [41]. The bactericidal effect was defined as MBC/MIC ≤4 [42]. Gentamicin served as the positive control.

### Statistical analysis

All analyses were performed in Stata Statistical Software Release 19 (StataCorp LLC, College Station, TX, USA). MIC and MBC values are presented as medians with interquartile ranges. Data distribution was assessed with the Shapiro–Wilk test. The Mann–Whitney U-test compared MIC and MBC values of betel extract in nanoemulsion versus DMSO against *Staphylococcus* and *P. aeruginosa*. The Kruskal–Wallis H test, followed by Dunn’s test with Bonferroni correction, was used to assess differences among the three CBD formulations. p < 0.05 was considered statistically significant.

## RESULTS

### GC-MS chemical composition of betel leaf extract

GC-MS analysis revealed that eugenol was the predominant compound in the betel leaf extract, representing 40.86% of the total peak area, followed by hydroxychavicol at 26.44% (Table 3). Other components were present in smaller proportions, including γ-muurolene (6.84%), δ-cadinene (3.56%), eugenol acetate (2.90%), chavicol (2.68%), trans-caryophyllene (2.20%), β-selinene (2.20%), 7-epi-α-selinene (1.62%), cis-calamenene (1.57%), copaene (1.07%), and α-humulene (1.06%). Unspecified minor constituents accounted for 7.00% of the chromatogram. The dominance of eugenol and hydroxychavicol is notable, as both compounds are recognized for their strong antibacterial activity in previous studies.

**Table 3:** Chemical composition of betel leaf extract analyzed by GC-MS.

Compound	Retention time	Kovat index	Percentage peak
Chavicol	9.876	1,250	2.68
Eugenol	11.682	1,359	40.86
Copaene	11.880	1,376	1.07
Trans-caryophyllene	12.539	1,419	2.20
Hydroxychavicol	12.973	1,424	26.44
α-Humulene	13.023	1,454	1.06
γ-Muurolene	13.216	1,479	6.84
β-Selinene	13.466	1,490	2.20
7-epi-α-Selinene	13.544	1,522	1.62
Eugenol acetate	13.648	1522	2.90
δ-Cadinene	13.755	1,523	3.56
cis-Calamenene	13.827	1,528	1.57
Others component			7.00

GC-MS = Gas chromatography-mass spectrometry

### MIC and MBC values of betel leaf extract nanoemulsion

The nanoemulsion containing 1 g/L of betel leaf extract was initially evaluated for minimum inhibitory dilution (MID) against *S. aureus* ATCC 6538 and methicillin-susceptible *S. pseudintermedius* (MSSP). The formulation demonstrated antibacterial activity against both strains, comparable to the nanoemulsion blank, with a MID of 1:4. To further enhance antibacterial effects, a higher concentration (5 g/L) nanoemulsion was subsequently prepared and tested against clinical *Staphylococcus* isolates and *P. aeruginosa*.

**Figure 1:**
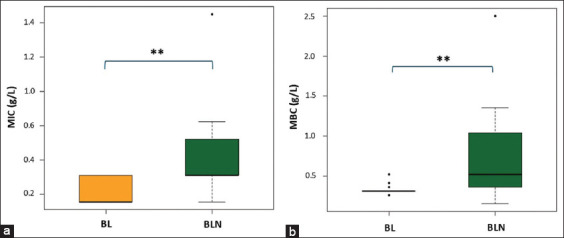
Box and Whisker plot showing Mann–Whitney U-test for the MIC (a) and MBC (b) of 5 g/L betel leaf extract (BL) in DMSO and nanoemulsion (BLN) against clinical *Staphylococcus* strains. The MIC and MBC of the betel leaf extract nanoemulsion were significantly greater than those in DMSO (**p ≤ 0.01). MIC = Minimum inhibitory concentration, MBC = Minimum bactericidal concentration, DMSO = Dimethyl sulfoxide.

The antibacterial efficacy of the betel leaf extract, at a concentration of 5 g/L in both its nanoemulsion formulation and dissolved in DMSO, was evaluated against *Staphylococcus* isolates using the Mann–Whitney U-test (Figure 1).

The betel leaf extract nanoemulsion exhibited potent antibacterial effects, with median MIC and MBC values of 0.31 g/L and 0.52 g/L, respectively. These values were significantly higher than those for the DMSO-dissolved extract, which demonstrated a median MIC of 0.16 g/L (p = 0.008) and MBC of 0.31 g/L (p = 0.007). The calculated MBC/MIC ratio for the nanoemulsion was less than 4, confirming a bactericidal mechanism of action [42]. For *P. aeruginosa* (n = 5), the median MIC of the betel leaf extract nanoemulsion (0.625 ± 0.42 g/L) was not significantly different from that of the DMSO preparation (0.625 ± 0.00 g/L) (p = 0.5556).

### MIC and MBC values of CBD formulations

**Figure 2:**
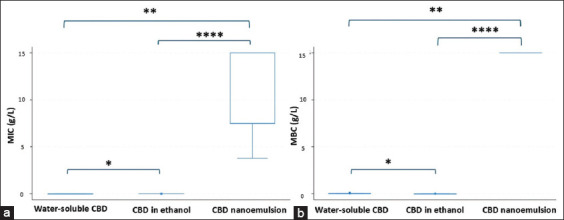
Box and Whisker plot showing Kruskal–Wallis H test for MIC (a) and MBC (b) of CBD in three different formulations (water-soluble CBD, CBD nanoemulsion, and CBD in ethanol) against clinical *Staphylococcus* strains. The results indicated that the MIC and MBC of the three formulations were significantly different (*p ≤ 0.05, **p ≤ 0.01 and ****p ≤ 0.0001). MIC = Minimum inhibitory concentration, MBC = Minimum bactericidal concentration, CBD = Cannabidiol.

The antibacterial efficacy of three CBD formulations, water-soluble CBD, ethanol-dissolved CBD, and CBD nanoemulsion, was evaluated against *Staphylococcus* isolates using the Kruskal–Wallis H test (Figure 2).

- MIC values: Median MICs were 0.003 g/L for ethanol-dissolved CBD, 0.004 g/L for water-soluble CBD, and 7.50 g/L for CBD nanoemulsion. Statistical analysis revealed significant differences among the formulations (p = 0.0001). Multiple comparisons revealed that the MIC of the nanoemulsion was significantly higher than that of both water-soluble CBD (p = 0.0003) and ethanol-soluble CBD (p < 0.0001). Furthermore, water-soluble CBD exhibited a higher MIC than ethanol CBD (p = 0.0343).
- MBC values: The corresponding median MBCs were 0.006 g/L, 0.31 g/L, and 15.0 g/L, respectively. Significant differences were observed (p = 0.0001), with CBD nanoemulsion displaying a markedly higher MBC than both water-soluble CBD (p = 0.0005) and ethanol CBD (p < 0.0001). Water-soluble CBD also had a significantly higher MBC compared with ethanol CBD (p = 0.0145).

The ethanol-based CBD exhibited an MBC/MIC ratio of 4, confirming bactericidal activity [42].

### Activity against Gram-negative bacteria

The antibacterial potential of the CBD formulations was further evaluated against Gram-negative bacteria. At tested concentrations, ethanol-dissolved CBD (0.10 g/L), water-soluble CBD (2.00 g/L), and CBD nanoemulsion (15 g/L) showed no inhibitory effect against *P. aeruginosa* ATCC 27853 or *E. coli* ATCC 8739. These results highlight the selective activity of CBD formulations against Gram-positive bacteria.

## DISCUSSION

### Antibacterial activity of betel leaf extract nanoemulsion

Although nanoemulsion technology has shown promise in drug delivery, its use in veterinary applications for natural plant compounds remains limited. This study developed a prototype antiseptic nanoemulsion containing betel leaf extract as a plant-based alternative for treating canine pyoderma caused by *Staphylococcus* spp. [43]. The nanoemulsion demonstrated strong antibacterial activity against MRSP, MSSP, and MRSS, with a median MIC of 0.31 g/L – well below the final product concentration of 5 g/L. This indicates high potency at relatively low concentrations, potentially minimizing adverse effects [44]. The extract also retained its bactericidal activity, as shown by a median MBC of 0.52 g/L and an MBC/MIC ratio of 4 [42]. Such bactericidal action is valuable for managing severe infections and in immunocompromised hosts [45]. While previous study by Valle *et al*. [46] reported non-toxic effects of lower extract concentrations on human skin cells, further investigation is needed to confirm the safety of the 5 g/L nanoemulsion in canine skin.

### Comparative efficacy and literature context

The nanoemulsion displayed slightly higher MIC values compared with the DMSO-dissolved extract, likely influenced by factors such as formulation, viscosity, and droplet size [47, 48]. The literature reports variable antibacterial outcomes with essential oil nanoemulsions. For instance, Ozogul *et al*. [49] observed greater activity of laurel essential oil compared with its nanoemulsion against *S. aureus* and *Enterococcus faecalis*, whereas Yazgan [50] reported that sage essential oil nanoemulsion showed superior antibacterial activity. The present study extended testing to the Gram-negative bacterium *P. aeruginosa*. The betel leaf extract nanoemulsion displayed strong activity, with a low MIC of 0.62 g/L. This is clinically relevant given the role of *P. aeruginosa* in deep skin infections, chronic otitis externa, and its emergence as a multidrug-resistant pathogen [1]. Optimizing the formulation and testing across diverse *P. aeruginosa* strains are warranted for clinical translation.

### Correlation of phytochemicals with antibacterial activity

This study linked the phytochemical composition of betel leaf extract to its antibacterial activity. GC-MS analysis identified eugenol (40.86%) and hydroxychavicol (26.44%) as the dominant constituents, consistent with earlier reports [23]. These phenolic compounds are well known for disrupting bacterial membranes and contributing to strong antibacterial effects [51, 52]. Establishing their quantified levels also provides a benchmark for quality control in future nanoemulsion production. Interestingly, the higher MIC observed in the nanoemulsion may reflect the limited water solubility of these compounds [53, 54]. Nonetheless, the nanoemulsion format offers several advantages, including improved stability, enhanced skin penetration, hydration benefits, and reduced pungency [55–57]. Beyond antibacterial effects, betel leaf extract also exhibits anti-inflammatory [58], antioxidant [59], and skin-safe properties [60], further supporting its potential as a topical therapeutic agent.

### Antibacterial efficacy of CBD formulations

The study also evaluated three CBD formulations, ethanol-dissolved, water-soluble, and nanoemulsion, against *Staphylococcus* spp. A pronounced difference in efficacy was observed. Ethanol-dissolved CBD and water-soluble CBD demonstrated potent inhibitory activity, with median MICs of 0.003 and 0.004 g/L, respectively. These findings align with earlier reports of CBD activity against *S. pseudintermedius* (0.003–0.012 g/L) [61], *S. aureus* (0.001 g/L), and *S. epidermidis* (0.002 g/L) [62]. Such values are comparable to chlorhexidine MICs (0.0012–0.00141 g/L) [23, 63], highlighting CBD’s potential as a topical alternative. The antibacterial mechanism is attributed to disruption of bacterial membranes and cell walls [64].

In contrast, the CBD nanoemulsion showed markedly reduced activity, with a median MIC of 7.50 g/L. This likely reflects the poor water solubility of CBD, limiting its availability to interact with bacterial cells [65]. The superior performance of the water-soluble formulation supports this interpretation and aligns with studies demonstrating enhanced antibacterial effects with improved solubility [66, 67].

### Spectrum of activity and safety considerations

None of the CBD formulations inhibited Gram-negative bacteria, including *E. coli* and *P. aeruginosa*, confirming the compound’s selective efficacy against Gram-positive species [68]. Importantly, previous studies by Lewińska [69] and Luz-Veiga *et al*. [70] indicate that topical CBD is generally safe, with low cytotoxicity to skin cells and no observed toxic effects. These findings suggest that CBD, especially in soluble forms, represents a promising candidate for further development as a safe and effective antiseptic in veterinary medicine.

## CONCLUSION

This study demonstrated the strong antibacterial potential of betel leaf extract nanoemulsion and CBD formulations against clinically relevant *Staphylococcus* isolates from canine pyoderma. The betel leaf extract nanoemulsion exhibited potent activity against MRSP, MSSP, and MRSS, with median MIC and MBC values of 0.31 g/L and 0.52 g/L, respectively, indicating bactericidal properties. It also inhibited *Pseudomonas aeruginosa* at a median MIC of 0.62 g/L, highlighting a broader antibacterial spectrum. GC-MS analysis identified eugenol (40.86%) and hydroxychavicol (26.44%) as the predominant phytochemicals, directly linking the extract’s chemical composition to its antimicrobial efficacy.

Among the CBD formulations, ethanol-dissolved and water-soluble CBD showed remarkably low MICs (0.003–0.004 g/L) against *Staphylococcus* spp., comparable to chlorhexidine, supporting their potential as powerful antiseptic alternatives. In contrast, CBD nanoemulsion was significantly less effective (MIC 7.50 g/L), likely due to poor solubility and limited bacterial interaction. None of the CBD formulations inhibited Gram-negative bacteria, consistent with their selectivity for Gram-positive species.

These findings have important practical implications, suggesting that betel leaf extract nanoemulsion and soluble CBD could serve as natural, plant-based alternatives to chlorhexidine for the topical treatment of canine pyoderma, reducing reliance on conventional antiseptics and supporting antimicrobial stewardship within a One Health framework. The additional anti-inflammatory and antioxidant properties of betel leaf extract further enhance its potential as a topical therapeutic. The study is strengthened by its use of clinical isolates, integration of phytochemical profiling with antibacterial assays, and identification of active compounds that may serve as quality-control markers for standardized formulations.

Nevertheless, some limitations remain. The findings are based on *in vitro* assays, and *in vivo* safety, skin penetration, and therapeutic efficacy need to be confirmed. The sample size of bacterial isolates was modest, and the underperformance of CBD nanoemulsion highlights formulation challenges that require further optimization.

Future studies should therefore focus on *in vivo* evaluations, formulation improvements to enhance solubility and delivery, testing against a wider range of multidrug-resistant Gram-negative pathogens, and exploring synergistic combinations of betel leaf extract and CBD.

This study provides compelling evidence that betel leaf extract nanoemulsion and soluble CBD formulations have significant potential as safe and effective topical antiseptics for canine pyoderma, particularly against multidrug-resistant *Staphylococcus* spp. Their adoption could reduce dependence on synthetic antiseptics such as chlorhexidine, mitigate the risk of AMR, and contribute to the advancement of natural product–based veterinary therapeutics.

## AUTHORS’ CONTRIBUTIONS

CP, PU, and NT: Conception and design of the study. WW, CP, and WS: Laboratory analysis. WW, CP, PU, NT, US, ST, and WS: Conducted the study and analyzed and interpreted the data. WW, CP, and ST: Conducted the assessment and interpreted the results. WW, CP, PU, and NT: Drafted the manuscript. WW, CP, PU, NT, US, and ST: Critically reviewed and revised the manuscript. All authors have read and approved the final manuscript.
